# Super‐killer CTLs are generated by single gene deletion of Bach2

**DOI:** 10.1002/eji.202249797

**Published:** 2022-09-26

**Authors:** Philippa R. Barton, Alexander J. Davenport, Jens Hukelmann, Doreen A. Cantrell, Jane C. Stinchcombe, Arianne C. Richard, Gillian M Griffiths

**Affiliations:** ^1^ Cambridge Institute for Medical Research University of Cambridge Cambridge Biomedical Campus Cambridge CB2 0XY UK; ^2^ Cancer Research UK Cambridge Institute University of Cambridge Cambridge Biomedical Campus Cambridge CB2 0RE UK; ^3^ Cell Signalling and Immunology Division, School of Life Sciences University of Dundee Dundee DD1 5EH UK

**Keywords:** CTL, BACH2, cytotoxicity, perforin, granzymes

## Abstract

*Bach2* codes for a transcriptional regulator exerting major influences on T cell‐mediated immune regulation. Effector CTLs derived from in vitro activation of murine CD8^+^ T cells showed increased proliferative and cytolytic capacity in the absence of BACH2. Before activation, BACH2‐deficient splenic CD8^+^ T cells had a higher abundance of memory and reduced abundance of naïve cells compared to wild‐type. CTLs derived from central memory T cells were more potently cytotoxic than those derived from naïve T cells, but even within separated subsets, BACH2‐deficiency conferred a cytotoxic advantage. Immunofluorescence and electron microscopy revealed larger granules in BACH2‐deficient compared to wild‐type CTLs, and proteomic analysis showed an increase in granule content, including perforin and granzymes. Thus, the enhanced cytotoxicity observed in effector CTLs lacking BACH2 arises not only from differences in their initial differentiation state but also inherent production of enlarged cytolytic granules. These results demonstrate how a single gene deletion can produce a CTL super‐killer.

## Graphical abstract

CTLs generated from mice lacking BACH2 show increased target cell killing. This arises both from increased numbers of memory CD8^+^ (CD8+mem) relative to naïve (CD8+n) splenocytes as well as the increased expression of cytolytic proteins localised in enlarged granules. This study shows that loss of BACH2 generates “super‐killer” CTLs.

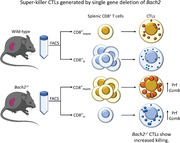

## 1 Introduction

Cytotoxic T Lymphocytes (CTLs) play a critical role in the immune system by eliminating virally infected and cancer cells. With the recent successes of immunotherapies that harness the killing capacity of CTLs, a better understanding of the many genetic regulators of CTL effector function becomes increasingly important.

How best to improve CTL killing has focused on increasing the recognition of target cells, either by altering the CTL itself [1, 2] or sensitizing their targets [3, 4]. CRISPR screens have been able to identify genes that increase the proliferative capacity or membrane targets of T cells that ultimately enhance cancer cell killing [5, 6]. However, to date, few specific genes impacting CTL cytolytic activity have been identified.

Using a large‐scale immunophenotyping screen of murine single gene deletion lines, we identified *Bach2* ^−/−^ CTLs as exhibiting an increased cytolytic capacity [7]. Previous studies of this transcription factor have shown that it prevents terminal differentiation to effector cells by limiting the expression of TCR‐driven genes and by increasing the generation of memory cells [8, 9, 10]. BACH2 is highly expressed in naïve CD8^+^ T cells and is downregulated upon activation, allowing the expression of genes required for effector cell development and function [11, 12], including *Gzmb, Prf1*, and *Fasl*, with ChIP Seq data consistent with binding at these gene loci [10]. Functional in vivo studies have demonstrated that *Bach2* ^−/−^ T cells were less efficient at clearing *Listeria monocytogenes* and inducing colitis after transfer of into *Rag1* ^−/−^ mice [13]. During chronic viral infection BACH2‐deficiency impaired stem‐like CD8^+^ differentiation [14]. Furthermore, *Bach2* ^−/−^ mice cleared tumors more efficiently than wild‐type [10]. Although these in vivo studies reveal functional differences in T cell responses, whether BACH2‐deficiency causes cell‐intrinsic changes in effector CTLs that might disrupt CTL‐mediated killing remained unclear.

In order to address this question, we compared wild‐type (WT) and BACH2‐deficient CD8^+^ T cells ex vivo and after in vitro effector differentiation. We found that *Bach2* ^−/−^ splenocytes contained a higher proportion of central memory T cells (T_CM_). After in vitro stimulation to generate cytolytic effector CTLs, those derived from T_CM_ showed enhanced killing compared with effector CTLs derived from naïve CD8^+^ T cells, regardless of *Bach2* genotype. Nevertheless, BACH2‐deficient effector CTLs derived from both naïve and T_CM_ populations killed better than their WT counterparts. In effector CTLs derived from both naïve and T_CM_ populations, we found an increased abundance of cytolytic proteins and larger cytolytic granules in *Bach2* ^−/−^ cells compared to WT controls. Thus, *Bach2* emerges as a single gene whose deletion gives rise to CTL “super‐killers,” both by increasing the proportion of memory‐phenotype cells at steady state, and by increasing the size and lytic protein content of CTL granules.

## 2 Results

### 2.1 *Bach2* ^−/−^ CTLs exhibit increased cytolytic and proliferative capacity

**Figure 1:**
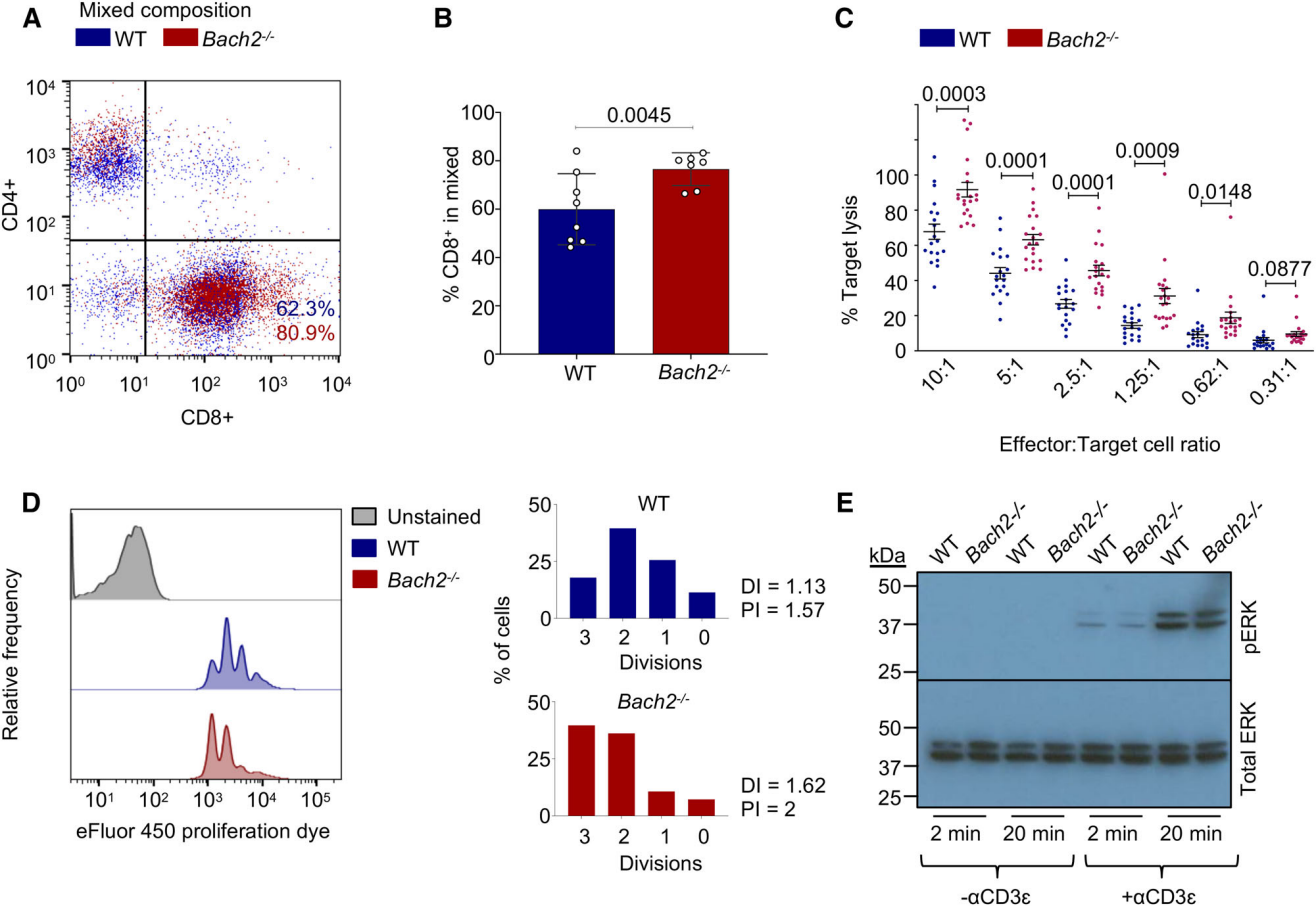
BACH2‐deficient CTLs show enhanced cytotoxicity. (**A**) Flow cytometry analysis showing percentage CD4^+^ and CD8^+^ T cells from WT or BACH2‐deficient splenocytes at day 8 post‐stimulation, representative of 7 (WT) or 8 (KO) independent experiments. (**B)** Bar chart summarizing repeats from (A). Symbols represent one individual mouse and bars indicate mean ± SD. **C** LDH release cytotoxicity assay of WT and BACH2‐deficient effector CTLs (8 days after activation) challenged with P815 target cells and anti‐CD3. Plots show the percentage of target cell lysis at 3 hours with CTL:target ratios as indicated from 20 BACH2‐deficient and 19 WT mice. Error bars indicate the mean and SD. Analysis performed as an unpaired *t*‐test. **D** Proliferation assay at 2 days post‐stimulation showing the proportion of total CD8^+^ T cells within each division peak, represented in corresponding bar charts with DI division index (DI), and proliferation index (PI). 2 independent repeats using 2 or 3 mice. (**E**) Western Blot showing phospho‐ERK1/2 and total ERK1/2 (44/42 kDa) with or without activation via anti‐CD3ɛ for 2 or 20 min. Three independent experiments.

*Bach2* ^−/−^ and WT splenocytes were activated on anti‐ CD3ɛ and CD28 coated plates and expanded for 8 days before measuring cytolytic capacity of the resulting effector CTLs in an LDH release assay of cytotoxicity. After activation, *Bach2* ^−/−^ samples contained a higher proportion of CD8^+^ T cells (Fig. 1A and B) and exhibited a marked increase in cytotoxic activity compared to WT controls across varying CTL to target ratios (Fig. 1C). Using a cytotoxicity assay that measures killing over time (Fig. S1A) showed that *Bach2^−/−^* effector CTLs generated either by stimulation of total splenocytes or purified CD8^+^ T cells exhibited greater lysis of target cells compared to WT controls at each time point until maximal killing was reached at 5 h. In the absence of BACH2, CD8^+^ T cells showed increased proliferation with a higher number of cellular divisions compared to WT controls after 2 days stimulation (Fig. 1D). As the strength of TCR signaling is directly linked to the effector function of CTLs, we investigated whether *Bach2* ^−/−^ CTLs exhibited differences in downstream signaling. TCR signaling has been shown to activate the MAPK pathway through the mediator ERK1/2 and phosphorylation of ERK1/2 indicates active TCR signaling. Activating WT and BACH2^−/−^ CTLs for 2 or 20 minutes with anti‐CD3ε, showed no difference in phospho‐ERK1/2 levels (Fig. 1E), suggesting that intracellular signaling is unchanged. These results show that while signaling was unchanged, *Bach2* ^−/−^ CTLs had increased capacity for killing targets compared to WT CTLs when measured either at varying CTL to target ratios or over time, suggesting a cell‐intrinsic enhancement of killing in the absence of BACH2.

### 2.2 *Bach2* ^−/−^ CD8^+^ T cells show an increased shift to effector phenotypes

**Figure 2:**
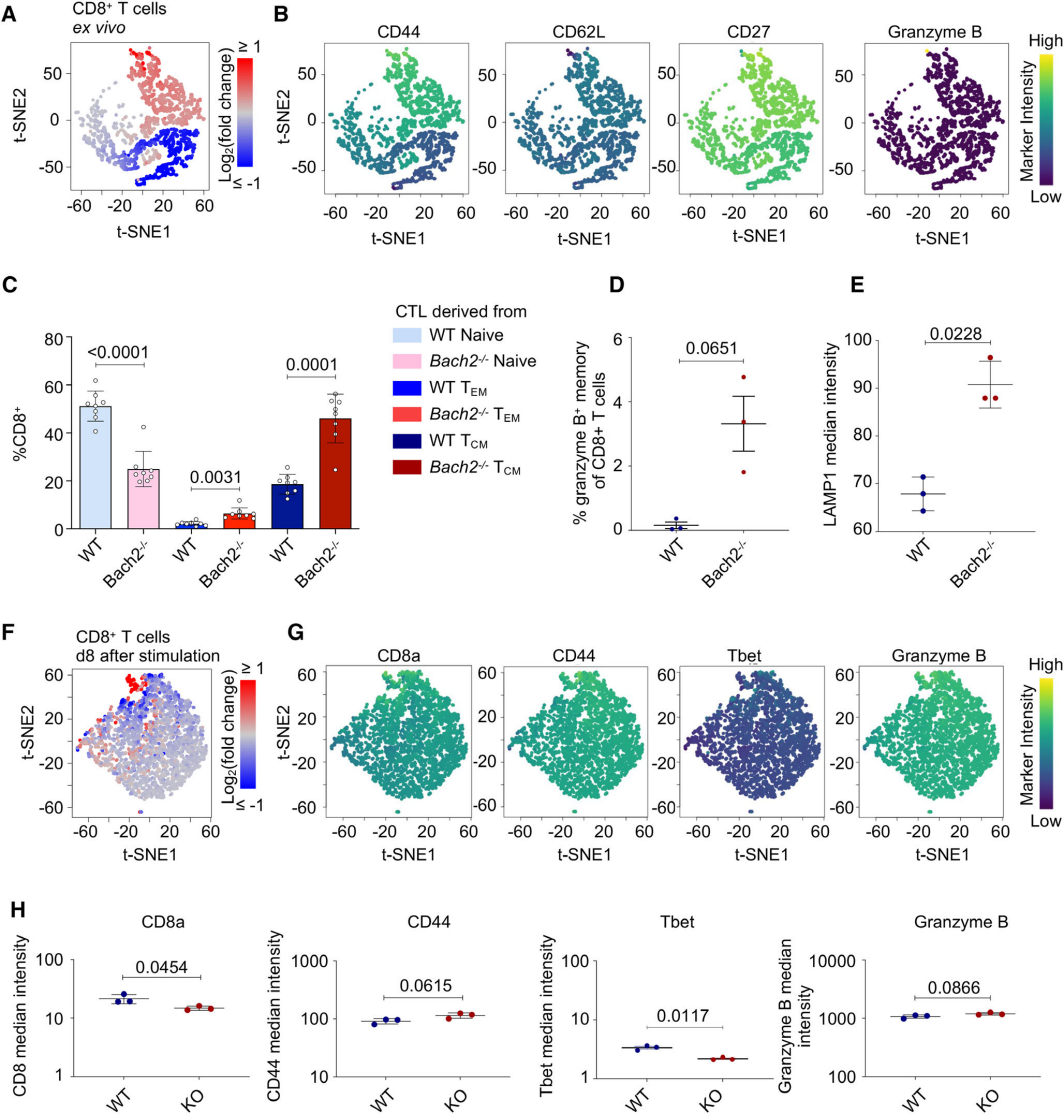
BACH2 deficiency increases the abundance of CD8^+^ T cells with activated phenotypes. (**A**) Protein expression was measured in ex vivo splenocytes from BACH2‐deficient and WT mice by mass cytometry (3 per genotype). Cells were gated on single, live CD45^+^ TCRβ^+^, CD8^+^ T cells before fine‐grained cell populations were identified by multi‐dimensional protein expression characteristics using cydar analysis (see Materials and Methods). The abundance of each of these populations was compared between BACH2‐deficient and WT mice. Each dot on the tSNE plot represents a differentially abundant population, colored according to populations that are more (red) or less (blue) prevalent in BACH2‐deficient samples compared to WT. (**B**) Expression of selected markers that define the populations from (A). Marker intensity depicts high (yellow) or low (purple) expression of the indicated marker. The full set of markers is depicted in Figure S3. (**C**) Naïve (CD62L^high^ CD44^low^), central memory (CD62L^high^ CD44^high^), and effector memory (CD62L^low^ CD44^high^) populations of CD8^+^ T cells were quantified in ex vivo splenocytes from Bach2^−/−^ and WT mice by flow cytometry. (**D**) Percentage of Granzyme B^+^ cells among CD8^+^ T cells, from mass cytometry data in (A). (**E**) Median signal intensity of LAMP1 in CD8^+^ T cells, from mass cytometry data in (A).  (**F**) As A for the same samples after 8 days of in vitro effector CTL differentiation. **G** Expression of selected markers that define the populations from F. The full set of markers is depicted in Figure S6. (**H**) Median signal intensity of indicated markers in CD8^+^ T cells, from mass cytometry data in (F). *p*‐values from unpaired *t*‐test.

We next used mass and flow cytometry to delineate broad immune cell types and specific phenotypes of CD8^+^ T cells and test for differential abundance between *Bach2^−/−^* and WT mice.  We first looked at *ex vivo* splenocytes (Figs. S2–S4). The B cell fraction of CD45^+^ splenocytes was reduced in *Bach2* ^−/−^ mice, as previously noted [15]. Conversely, myeloid cells were more abundant in the absence of BACH2. Gating on CD8^+^ T cells showed that BACH2 deficiency leads to a reduction in naïve and an increase in memory CD8^+^ T cells (Fig. 2A–C; Fig. S3). Of note, a small population of memory CD8^+^ T cells (CD44^high^) expressed high levels of the cytolytic granule component granzyme B, and these were upregulated in *Bach2* ^−/−^ mice (Fig. 2B and D). LAMP1 (CD107A), which is a transmembrane protein of the cytolytic granules, was increased in *Bach2* ^−/−^ samples, correlating with the increased abundance of effector‐phenotype T cells (Fig. 2E).

To identify the differences in CTLs that might be affecting their intrinsic cytolytic capacity, we differentiated effector CTLs from *Bach2* ^−/−^ and WT splenocytes for 8 days and again compared their phenotypes. After this in vitro differentiation, *Bach2* ^−/−^ samples exhibited a slightly higher percentage of CD8^+^ T cells and a concomitant reduction of CD4^+^ T cells (Figs. S5–S7 and Fig. 1B and C). Gating on the CD8^+^ T cells revealed WT and *Bach2^−/−^* effector CTLs with a similar expression of most proteins measured (Fig. 2F; Fig. S6). Subtle differences in the absence of *Bach2* ^−/−^ were characterized by slightly reduced CD8 and T‐bet expression and a trend toward increased CD44 and Granzyme B (Fig. 2G and H). In addition, the increased prevalence of a subpopulation of CD8^+^ T cells stained by Gr‐1 antibodies suggests upregulation of Ly6C in a small subset of *Bach2* ^−/−^ cells (Fig. S6). Together, our results show an increased tendency toward effector phenotypes in *Bach2^−/−^* CD8^+^ T cells, consistent with previous studies showing that BACH2 blocks effector T cell differentiation [8, 11, 12], but that under in vitro effector differentiation conditions, WT and *Bach2* ^−/−^ cells both become effector CTLs.

### 2.3 Bach2^−/−^ effector CTLs derived from naïve and T_CM_ populations exhibit increased cytolytic capacity

**Figure 3:**
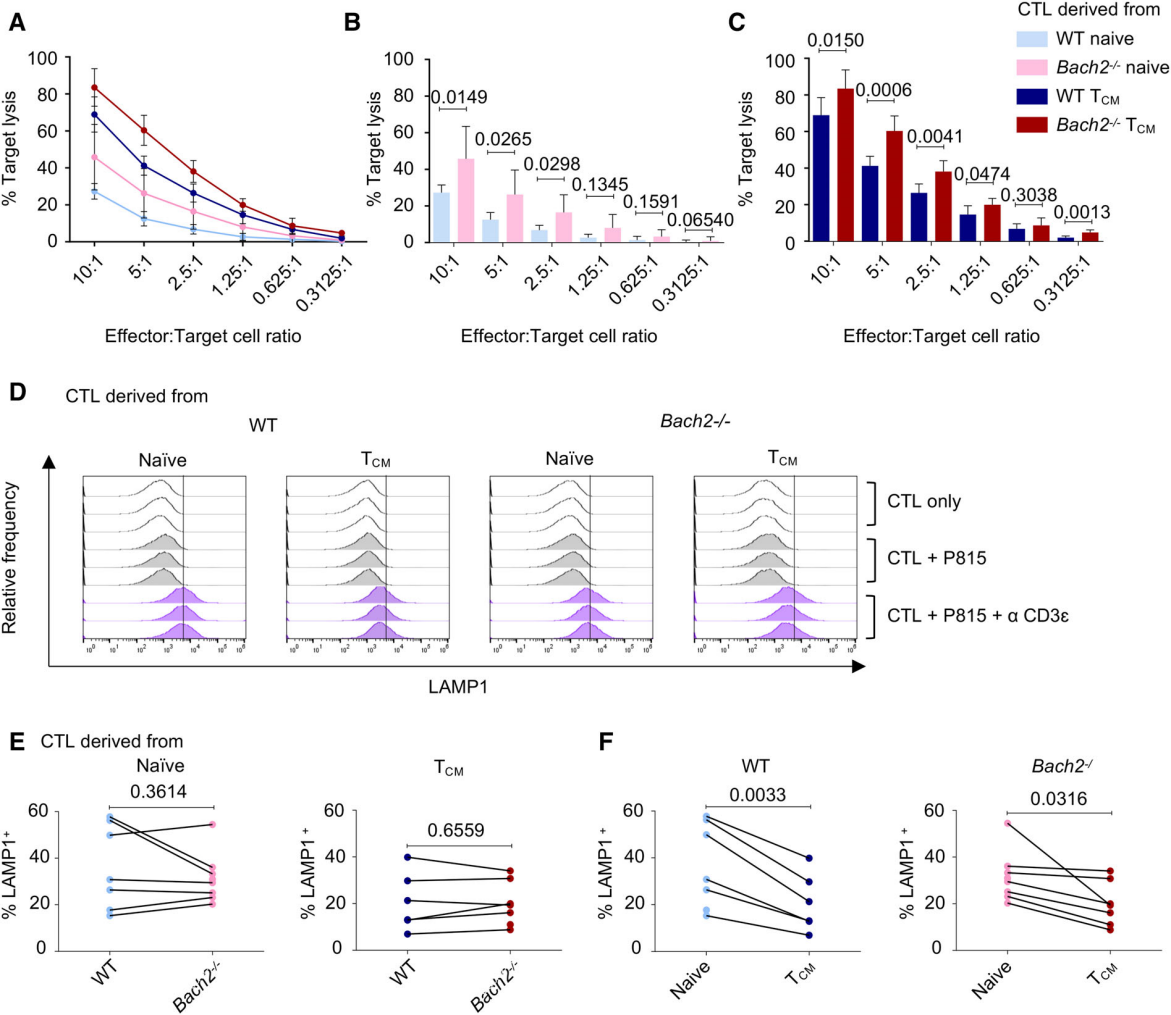
Pre‐stimulation differentiation state and BACH2 deficiency both impact cytotoxic potential in in vitro‐derived effector CTLs. (**A**) Combined data from LDH release cytotoxicity assays from four independent experiments, performed with a total of 7–8 mice per genotype, showing the percentage lysis of target P815 cells by WT and BACH2‐deficient CTLs, at the indicated E:T ratios. Error bars show SD from the mean of independent repeats. (**B**) Data from A comparing WT and BACH‐2‐deficient CTLs derived from the naïve population with bars indicating mean ± SD. Analysis by multiple unpaired *t*‐test. (**C**) As B for WT and BACH2‐deficient effector CTLs derived from T_CM_. (**D**) CTL degranulation ± P815 target cells ± αCD3ε after 2 h. Histograms show relative frequency for triplicate samples from WT and *Bach2* ^−/−^ CTLs derived from naïve cells or T_CM._ Vertical lines mark maximal LAMP1 expression without stimulation. Representative of 7 (WT) or 8 (KO) independent repeats with cells from two to three mice per pool. (**E** and **F**) Data from D with dots showing values from individual experiments. *p*‐Values from paired *t*‐test are shown.

As our data showed differences in the differentiation state of *ex vivo* splenic CD8^+^ T cells in *Bach2^−/−^* mice, we next separated naïve and T_CM_ populations prior to stimulation. Effector memory T cells (T_EM_) made up a small percentage of both WT and KO samples and failed to expand post stimulation. Effector CTLs derived from T_CM_ exhibited greater cytotoxicity compared to those derived from naïve T cells, but BACH2 deficiency still increased cytotoxicity, even within these purified populations. (Fig. 3A–C).

Despite greater cytotoxicity, *Bach2^−/−^* effector CTLs did not exhibit a difference in granule release in response to target cell recognition compared to WT samples (Fig. 3D and E). However, CTLs derived from naïve cells showed a statistically significant increase in degranulation compared to those derived from T_CM_, regardless of *Bach2* genotype (Fig. 3F).

### 2.4 Differentiation state and BACH2‐deficiency alter granule size in CTLs

**Figure 4:**
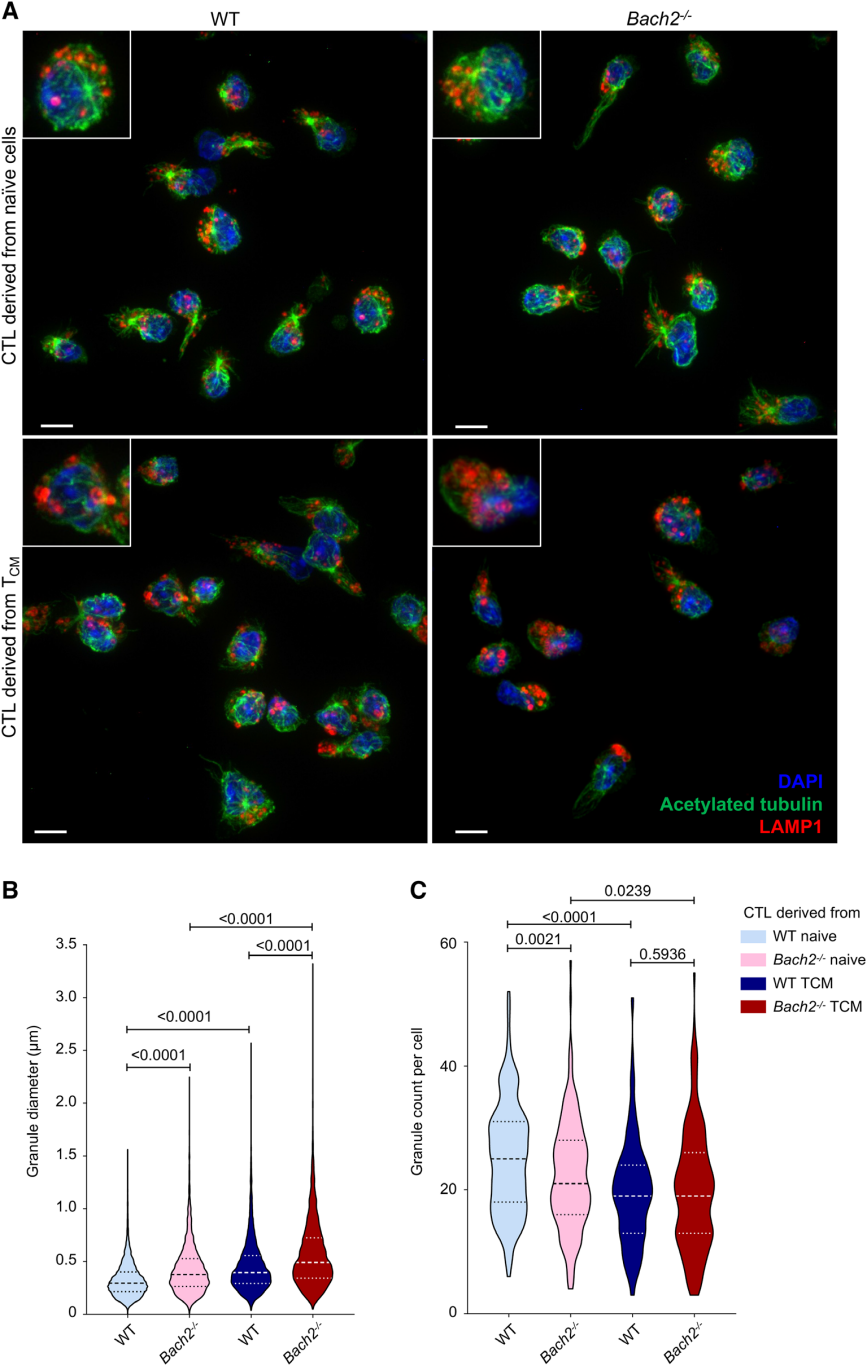
Both genotype and initial differentiation state alter the size and number per cell of lytic granules of in vitro‐differentiated effector CTLs. (A) WT and BACH2‐deficient effector CTLs derived from naïve cells or T_CM_ labeled with antibodies against tubulin, LAMP1 (lytic granules), and DAPI (nucleus). Scale bar = 5 μm. (B) Violin plots showing quantification of granule diameter from WT CTLs derived from naïve (n = 4520), or Tcm (n = 3516) or BACH2‐deficient CTLs derived from naïve (n = 3980) or Tcm (n = 3587). Data pooled from three independent repeats, with 2–3 mice per genotype per experiment. (**C**) Violin plots showing the quantification of number of granules per cell from 30 cells per condition counted in each independent repeat. WT cells stimulated from three independent experiments analyzing a total of 180 cells per genotype per condition (90 female, 90 male).

We next imaged the cytolytic granules in *Bach2^−/−^* versus WT effector CTLs by immunofluorescence, using DAPI and antibodies against LAMP1 and tubulin to identify nuclei, granules, and microtubules, respectively (Fig. 4A). We found that granules in BACH2‐deficient effector CTLs were larger than those in WT CTLs, and that granules in effector CTLs derived from T_CM_ were larger than those in CTLs derived from naïve cells (Fig. 4B). In effector CTLs derived from naïve cells, there were fewer granules per cell in *Bach2* ^−/−^ than WT, but there was no difference in effector CTLs derived from T_CM_ (Fig. 4C). Regardless of *Bach2* genotype, effector CTLs derived from naïve cells had more granules than those derived from T_CM_ (Fig. 4C). Thus *Bach2^−/−^* effector CTLs had larger granules than WT, and granules in effector CTLs derived from T_CM_ were larger but less numerous than those derived from naïve cells.

### 2.5 Enlarged cytolytic granules in *Bach2* ^−/−^ CTLs contain higher levels of lytic proteins

**Figure 5:**
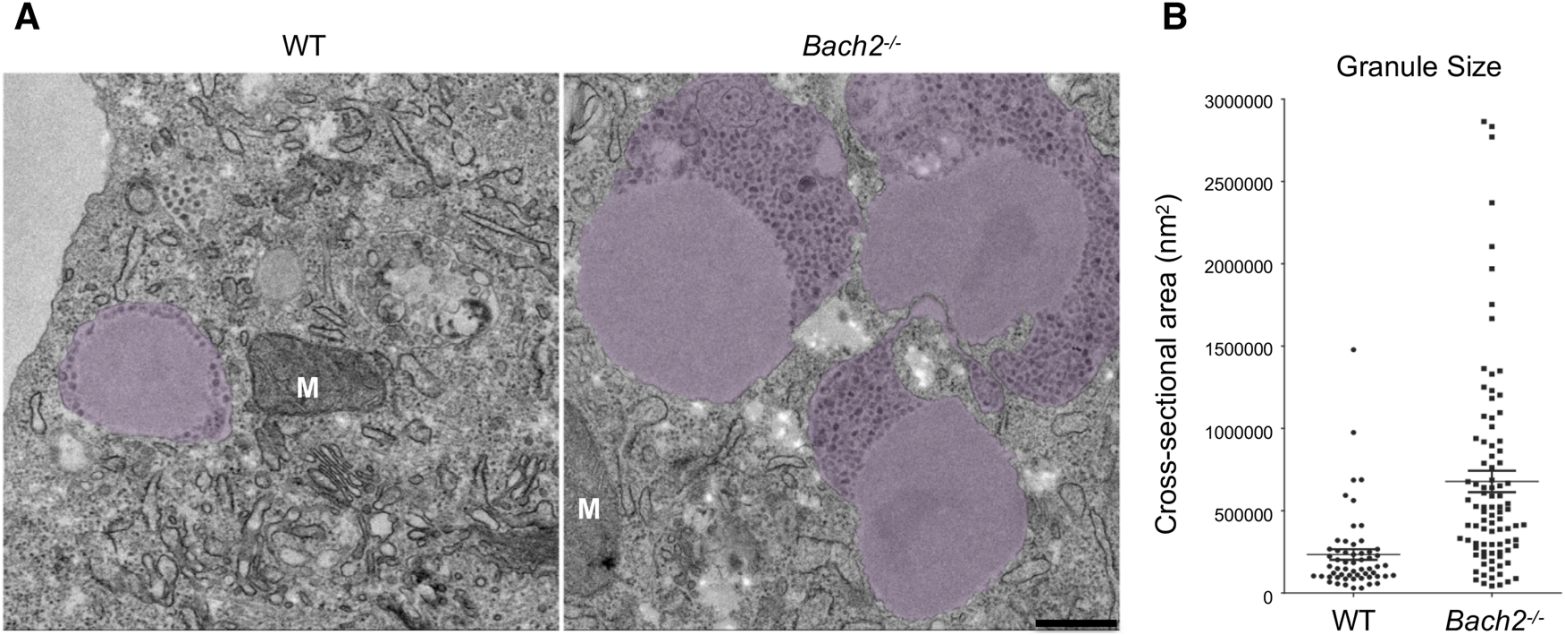
BACH2‐deficient effector CTLs contain enlarged lytic granules and have higher levels of lytic protein compared to WT. (**A**) Transmission electron micrograph (TEM) of CTLs from WT (left) and BACH2‐deficient (right) mice. False color indicates lytic granules; M, mitochondria. Scale bar = 500 nm (applies to both panels). (**B**) Cross‐sectional area of individual granules from WT (n = 58) and BACH2‐deficient (n = 88).

Cytolytic granules are made up of a dense secretory core surrounded by multivesicular structures, contained within an outer limiting membrane. The crystalline core contains lytic proteins including perforin and the family of granzymes [16, 17, 18]. In order to understand the changes in granules at the ultra‐structural level, we examined *Bach2^−/−^* and WT effector CTLs by transmission electron microscopy (TEM). While granule anatomy was largely unchanged in *Bach2* ^−/−^ effector CTLs, with dense core and multi‐vesicular regions similar to WT, the granules in *Bach2* ^−/−^ cells were considerably larger than their WT counterparts, with both the secretory core and surrounding multivesicular area enlarged (Figure 5).

**Figure 6:**
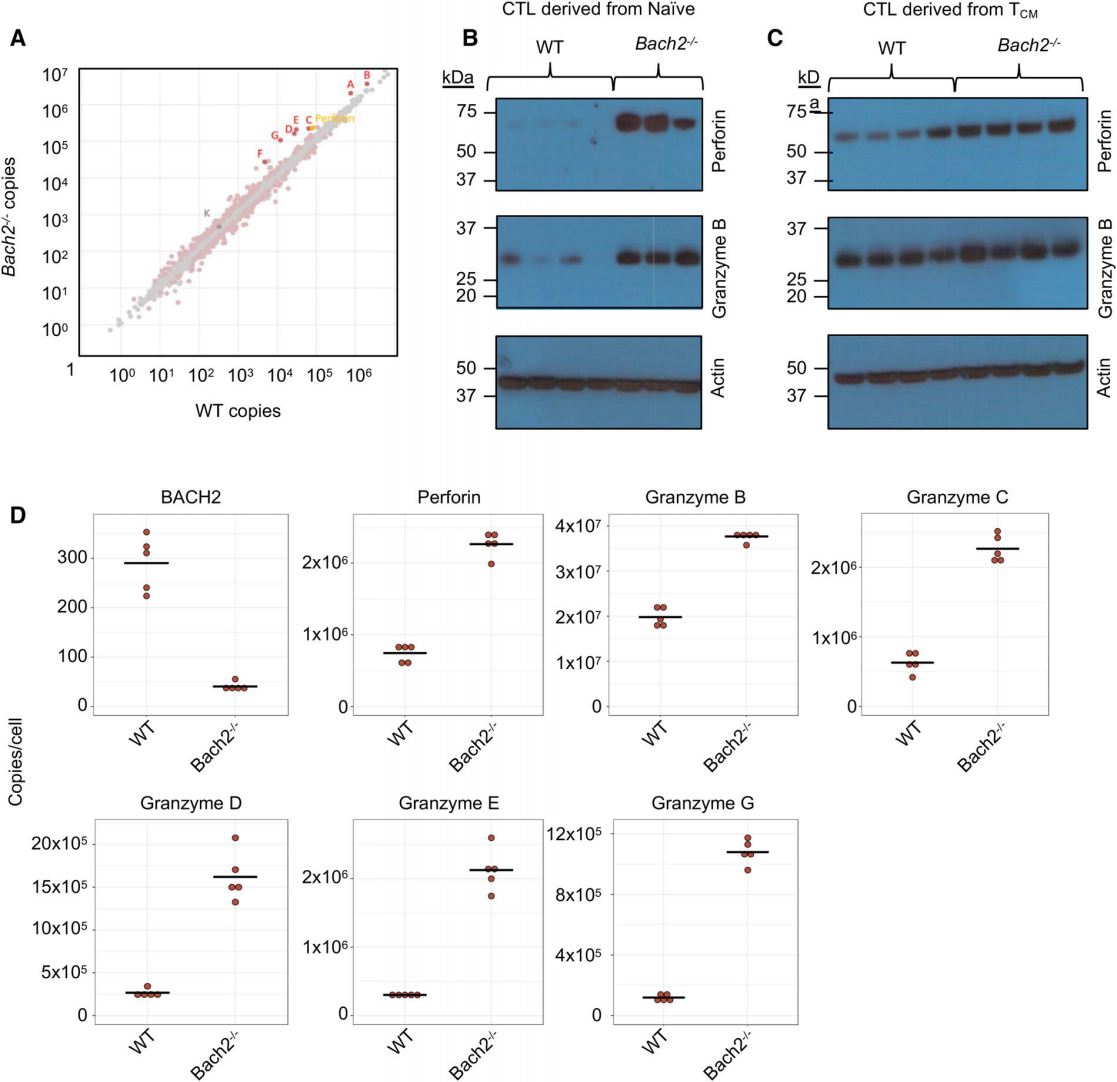
Differential expression analysis was performed on mass spectrometry data from BACH2‐deficient versus WT effector CTLs. (**A**) Copy number per protein in *Bach2* ^−/−^ versus WT CTLs, with CTL lytic proteins highlighted. For non‐highlighted proteins: pink = *p*‐value < 0.05 and grey = *p*‐value > 0.05. For highlighted proteins of interest: red = *p*‐value < 0.05 as well as *z*‐score > 2; yellow = *p*‐value < 0.05 as well as *z*‐score < 2 and grey = *p*‐value > 0.05 as well as *z*‐score < 2. Labels (A–G) and (K) indicate different Granzyme isoforms. (**B** and **C**) Immunoblots showing perforin and granzyme B protein expression in WT and BACH2‐deficient effector CTLs differentiated from naïve cells and T_CM_. WT n = 7, KO n = 7. (**D**) Quantification of proteomics analysis showing copies per cell for BACH2, Perforin, Granzyme B–E and G from WT and BACH2‐deficient CTLs.

In order to quantitate any differences in protein content, we examined the proteomes of *Bach2* ^−/−^ and WT effector CTLs. Differential expression analysis revealed significant differences in the expression of major cytotoxic granule proteins in *Bach2* ^−/−^ effector CTLs, including increased expression of perforin, and granzymes A‐G (serine proteases involved in target cell killing) (Fig. 6A). Higher expression of perforin and granzyme B in *Bach2* ^−/−^ compared to WT effector CTLs was also seen by Western blotting, most notably in CTLs derived from naïve cells (Fig. 6B), although the same trend was observed in those derived from T_CM_ (Fig. 6C). Highlighting the most significant changes in copy numbers from the proteomics confirmed the loss of BACH2 protein in the *Bach2* ^−/−^ CTLs, as well as the increased copy numbers of perforin and Granzymes B‐G (Fig. 6D).

## 3 Discussion

Recent studies have characterized the role of BACH2 in a number of immune cell types [19, 20, 21, 22, 23, 24]. However, changes in CTL killing have not been examined. We identified *Bach2* ^−/−^ in an unbiased screen of single gene deletion mice as giving rise to super‐killer CTLs with increased lytic capacity. Investigating the basis for this increased killing, we found that splenic CD8^+^ T cells from *Bach2* ^−/−^ mice contained increased proportions of T_EM_ and T_CM_. We found that although *Bach2* ^−/−^ effector CTLs killed better than their WT counterparts, there were no differences in pERK signaling or degranulation. However, *Bach2* ^−/−^ effector CTLs showed elevated levels of lytic proteins (granzymes and perforin) in enlarged granules, suggesting that each granule release might be more effective in killing target cells. This idea is supported by the imaging‐based killing assay that measures target cell killing over time, as *Bach2* ^−/−^ CTLs showed increased killing compared to WT at each time point until maximal killing was reached.

We noted that both *Bach2* ^−/−^ and WT effector CTLs derived from T_CM_ killed better than those derived from naïve cells, with higher expression of lytic proteins, larger granules, and less degranulation. These larger lytic granules with more lytic proteins may therefore be more capable of killing targets, either quickly through a single large hit of lytic protein, or through increased serial killing, resulting in more sustained killing. However, importantly, *Bach2* ^−/−^ effector CTLs always showed enhanced lytic capacity compared to their WT counterparts, regardless of whether they were derived from naïve or T_CM_. Thus, the loss of BACH2 generates super‐killer CTLs in our system, both by increasing the proportion of T_CM_ in unstimulated splenocytes and by increasing the inherent expression of lytic proteins and the size of the lytic granules.

With increasing use of CTLs in immunotherapies, there is great interest in finding mechanisms to enhance T cell killing. Many studies have focused on increasing the sensitivity of targets to CTL lysis, for example, by upregulating the expression of MHC class I on target cells [25, 26], or disrupting the cell cycle by radiation or WEE1 kinase inhibition [27, 28]. Other studies have aimed to enhance the lytic activity of CTLs, for example by combination therapies with PD‐1 blockade that increase CD8^+^ T cell count [29], or by increasing CTL tumor infiltration [30]. Although these approaches increase CTL cytotoxicity, none enhance the inherent lytic capacity of individual T cells.

In this study, we have demonstrated that the single gene deletion of *Bach2* enhanced CTL cytotoxicity. Although *Bach2* deletion affected many cell types when deleted in the germline, our study showed that changes induced by the loss of BACH2 shifted the differentiation of CD8^+^ T cells to memory‐like cells and also increased lytic protein production in *Bach2^−/−^* effector CTLs. Whether germline deletion is required for enhanced CTL killing remains an open question. In studies following the production of stem‐like CD8^+^ T cells, acute deletion of *Bach2* recapitulated the findings from germline deletion, indicating that many of the cell intrinsic effects seen with germline deletion could be generated via acute deletion [14]. Regardless, these findings suggest that it may be possible to generate CAR‐T cells with enhanced killing capacity by deletion of *Bach2* in inducible pluripotent stem cells (iPSCs) before differentiation into CTLs [31, 32, 33].

## 4 Materials and methods

### 4.1 Mice

CD8^+^ T cells were isolated from *Bach2* ^−/−^ (*Bach2* ^tm1a(EUCOMM) Wtsi^) and WT mice on the C57BL/6N genetic background. Mice were bred and housed in the University of Cambridge Facility. This research has been regulated under the Animals (Scientific Procedures) Act 1986 Amendment Regulations 2012 following ethical review by the University of Cambridge Animal Welfare and Ethical Review Body (AWERB).

### 4.2 Cell culture and stimulation

Mouse spleens were homogenized using a sterile syringe and a sterile 70μm nylon strainer to achieve a single cell suspension of splenocytes. Splenocytes were stimulated for 48 hours on plates pre‐coated with 0.5 μg/ml α‐CD3ε (500A2, eBioScience), 1 μg/ml α‐CD28 (37.51, eBioscience) and expanded for up to 1 week in CTL culture medium (RPMI 1640, 10% FBS, 2 mM glutamine, 1 mM sodium pyruvate, 100 U/ml penicillin, 1 mg/ml streptomycin, 50 μM β‐mercaptoethanol, 100 U/ml IL‐2) at 37°C. Functional assays were performed 8 days after stimulation unless otherwise stated. CD8^+^ T cells were isolated using the Mouse CD8a^+^ T cell Isolation Kit (Miltenyi Biotec) where required.

The target cell line, Mus musculus mastocytoma P815, was obtained through ATCC, and was cultured in DMEM (Sigma), 10% FBS (LabTech), and penicillin‐streptomycin (Sigma). P815 stably expressing the Nuclight Red Lentiviral vector (Essen) were cultured in the presence of puromycin (Invitrogen).

### 4.3 Cell sorting

Splenocytes were isolated from C57BL/6 WT and *Bach2* ^−/−^ (*Bach2^tm1a(EUCOMM) Wtsi^*) mice, pooling two spleens from mice of the same sex per biological replicate. Samples were enriched for CD8^+^ T cells, using the Mouse CD8α^+^ T cell Isolation Kit (Miltenyi Biotec). Cells were resuspended in PBS,1% FBS and stained with combinations of α‐CD8a conjugated to Bv711 or FITC, α‐CD44 conjugated to PE or APC, α‐CD62L APC, α‐CD44 (all from Biolegend) and α‐CD62L PE (eBioscience), together with the viability indicator, DAPI.

Cells were sorted on an Influx cell sorter (Becton Dickinson). FSC‐H and SSC‐H were used to exclude debris, doublets were excluded using FSC‐H and trigger pulse width, and dead cells were excluded using DAPI. CD8^+^ cells were sorted into Naïve (CD44^low^, CD62L^high^), T_EM_ (CD44^high^, CD62L^low^), and T_CM_ (CD44^high^, CD62L^high^) T cell subpopulations.

### 4.4 Flow cytometry

To assess T cell subsets after stimulation and expansion (Figure 1), cells were stained in PBS (Gibco) containing 1% FBS (LabTech) with α‐CD4 PE (Biolegend) and α‐CD8α APC (Biolegend) at day 8 post‐stimulation. Samples were analyzed on the FACSCalibur (BD Biosciences). Data were analyzed using FlowJo v10.

To assess proliferation splenocytes were incubated with Cell Proliferation Dye eFluor 450 (Thermo Scientific). Samples were taken prior to stimulation and at day 2 post‐stimulation using plate‐bound α‐CD3ε (eBioscience) and α‐CD28 (eBioscience). Cells were resuspended in PBS (Gibco) containing 1% FBS (LabTech) with FCR (Fc receptor) blocking antibody (Biolegend) and the Zombie‐Yellow Fixable Viability Kit (Thermo Scientific). Cells were analyzed using an LSR Fortessa (BD Biosciences). FSC‐H and SSC‐H were used to exclude debris, doublets were excluded using FSC‐H and trigger pulse width, and dead cells were excluded using Zombie Yellow. eFluor450 fluorescence intensity was measured to mark cell division. The division and proliferation index of samples was calculated using FlowJo v10.

To assess degranulation capacity (Fig. 3D–E), CTLs were re‐suspended in RPMI 1640 (Gibco) in the presence of PE‐conjugated α‐CD107a (α‐LAMP1, Biolegend) antibody and mixed with target P815 cells and α‐CD3ɛ (eBioscience) at an effector‐target‐ratio of 1:1. Unstained control wells were plated in the absence of α‐LAMP1 and unstimulated controls were plated in the absence of either α‐CD3ɛ (eBioscience) only or both target P815 cells and α‐CD3ɛ. Samples were incubated at 37°C for 2h before being stained in ice‐cold PBS (Gibco) with α‐CD8α APC (Biolegend) and live/dead marker DAPI (Thermo Scientific). Cells were analyzed on the Attune NxT (Thermo Scientific) or LSR Fortessa (BD biosciences) flow cytometers. Gating was determined by the single stain controls and the WT unstimulated controls for each time point. FSC‐H and SSC‐H were used to exclude debris, doublets were excluded using FSC‐H and trigger pulse width, and dead cells were excluded using DAPI (Thermo Scientific). Samples were gated on CD8^+^ cells using the CD8^−^ target P815 cells as a negative control. A final gate was set on cells positive for LAMP1 (Biolegend), using the unstimulated WT sample from each time point. Data were analyzed using FlowJo v10.

### 4.5 Mass cytometry

*Bach2* ^−/−^ and WT cells were examined by mass cytometry before and after stimulation and expansion. Mass cytometry antibodies are listed in Table S1. Conjugations of 155Gd‐α‐CD44 (clone IM7) and 160Gd‐α‐PE (clone PE001) were performed using Biolegend MaxPar Ready antibodies using the Maxpar Antibody Labelling Kit (Fluidigm), with 83 and 74 μg antibody, respectively.  Mass cytometry cell staining was performed with MaxPar kits (Fluidigm). Cell ID Cisplatin (0.25 μM) was used to stain live cells for 5 min at room temperature. Cells were then barcoded with the Cell‐ID 20‐Plex Pd Barcoding Kit and pooled. FC receptors were blocked with α‐mouse CD16/32 (clone 93, Biolegend), followed by staining with primary and then secondary antibodies in MaxPar Cell Staining Buffer. The Maxpar Nuclear Antigen Staining Buffer Set was used to fix and permeabilize cells before re‐blocking FC receptors and staining with intracellular primary and secondary antibodies. Cells were left overnight in 125 nM Cell‐ID Intercalator‐Ir in Maxpar Fix and Perm Buffer and then analyzed on a Helios CyTOF system (Fluidigm).

On Day 0 splenocytes were treated with RBC Lysis Buffer (eBioscience) to remove red cells before staining. Cells from days 0 and 8 were stained and analyzed separately. For both experiments, 3×10^6^ cells from each of 6 mice (3 *Bach2* ^−/−^, 3 WT) were stained for live cells and barcoded. 1.5 × 10^6^ barcoded cells from each sample were pooled for surface and intracellular staining.

### 4.6 Mass cytometry data analysis

Helios Software and Debarcoder (Fluidigm) were used to normalize and de‐barcode mass cytometry data. Differential abundance analyses were carried out using the cydar Bioconductor package [34] in R. The following filters were applied to cells pooled across all samples. Due to large datasets, a fifth of the acquisition time from Day 0 and a twentieth of the acquisition time from day 8 were used in differential abundance analyses. We then removed normalization beads, non‐singlet cells (dnaGate function on 191Ir and 193Ir channels), dead cells (195Pt high), and CD45^−^ cells (5 median absolute deviations below median). For analyses of just CD8a^+^ T cells, we further gated on TCR^+^ cells and CD8a^+^ CD4^−^ cells. On Day 8, strong Granzyme B staining (173Yb) appeared to spill over into neighboring perforin and IL‐7Ra channels (174Yb IL‐7Ra and 172Yb Perforin). We therefore excluded these two channels before analysis. A hypersphere radius with a tolerance of 0.5 was used for assigning cells to phenotypic hyperspheres and differential abundance was assessed by fitting a quasi‐likelihood negative binomial GLM in edgeR, with gender as a blocking factor, and performing quasi‐likelihood F‐tests [35]. Hyperspheres with significantly different cell abundances between *Bach2* ^−/−^ and WT samples were identified with spatial FDR controlled at 5%. t‐SNE plots were made using the Rtsne package [36].

### 4.7 LDH release cytotoxicity assay (fixed time points)

The enzyme Lactate dehydrogenase (LDH) is present in the cytoplasm of cells and, upon cell death, is released into the cytoplasm. The amount of LDH present is therefore representative of the amount of cell death that has occurred and can be quantified using The CytoTox 96 Non‐Radioactive Cytotoxicity Assay (Promega). CTLs at day 8 post‐stimulation were re‐suspended in RPMI (Gibco) containing 2% heat‐inactivated FBS (LabTech) and penicillin/streptomycin (Sigma) and mixed with P815 target cells at the effector‐to‐target ratios shown and α‐CD3ɛ (1 μg/ml), which is bound by Fc receptors on P815 targets and stimulates directly lytic activity by CTLs. Control wells included CTLs and P815 targets without α‐CD3ɛ. Cells were incubated at 37°C for 2 h and 15 min before the addition of lysis buffer (Promega) to positive control samples. Samples were incubated at 37°C for a further 45 min. After the full 3 h incubation, the percentage of target cell lysis was determined using the CytoTox 96 Non‐Radioactive Cytotoxicity Assay (Promega) following the manufacturer's instructions and quantified using either a SpectraMax plate reader (Molecular Devices) using the SoftMaxPro software (Molecular Devices) or Spark multimode microplate reader (Tecan), using the absorbance wavelength of 490nm. Percentage lysis was calculated as follows: (((CTL + P815 + α‐CD3ɛ) − (CTL + P815))/positive control) × 100).

### 4.8 Incucyte cytotoxicity assay (measuring killing over time)

P815 cells stably expressing a red nuclear marker (Essen) were seeded (±0.5 μg/ml α‐CD3ɛ (500A2, eBioscience)) in a round bottom, ultra‐low attachment 96‐well plate (Corning) and centrifuged to pellet. CTLs were seeded giving an E:T ratio of 10:1. Samples were centrifuged and incubated at 37°C and 5% CO_2_, in the Incucyte S3 live cell analysis system (Essen Bioscience). A 4× objective was used to capture images, the first ∼10 min after initial incubation and then once every hour. The spheroid quantification tool (IncuCyte S3 software) was used to measure the resulting change in fluorescence intensity of the target cell pellet over time. The percentage target cell lysis was calculated relative to the time point with the highest fluorescence Intensity reading (generally 0 or 1 h), to avoid negative target cell lysis due to initial target cell settling.

### 4.9 Immunoblotting

Cell pellets used to analyze pERK signaling were generated from CTLs at day 8 post‐stimulation. CD8^+^ T cells were isolated at day 6 post‐stimulation, using the Mouse CD8a T cell Isolation Kit (Miltenyi Biotec) and stimulated with 0.5 μg/ml plate‐bound α‐CD3ε (500A2 eBioscience) for 2 or 20 min. Cells were lysed immediately at 2 × 10^7^ cells/ml in 50 mM Tris‐HCL (pH 8), 150 mM NaCl, 1 mM MgCl_2_, 2% Triton X‐100, 1× protease inhibitor cocktail (Roche), and 1× Phos‐STOP (Sigma). Lysates were loaded onto a NuPAGE 4–12% Bis‐Tris gel in 10% NuPAGE Sample buffer with a reducing agent (Thermo Scientific) and run in 1× MES SDS running buffer at 110–130 V for 1–2 h. Precision Plus Protein Kaleidoscope MW markers were used (BioRad). Proteins were transferred onto nitrocellulose membranes using 1× NuPAGE transfer buffer, 10% methanol at 100 V for 95 min. The membrane was blocked in PBS, 0.05% Tween‐20, 5% dried milk (or 5% BSA for pERK blots). Antibodies were diluted into the respective blocking buffers with primary antibodies: α‐mouse β‐actin (Sigma); α‐mouse granzyme B (Abcam); α‐mouse perforin (Enzo), followed by goat α ‐rabbit IgG (Thermo Scientific), goat α‐mouse IgG (Thermo Scientific), or goat α‐rat IgG (Thermo Scientific) secondary antibody conjugated to HRP. Chemiluminescence was detected using ECL prime WB detection reagent (GE Healthcare). Image detection was achieved using either a ChemiDoc MP Imager (Bio‐Rad) or using an X‐ray film processor (EcoMax).

### 4.10 Immunofluorescence imaging

Cells were seeded in prewarmed CTL culture medium for 10 min at 37°C. Cells were fixed in ice‐cold methanol (Thermo Scientific) for 5 min. Slides were blocked in PBS (Gibco) containing 1% BSA for 25 min. Primary antibodies, mouse anti‐alpha‐tubulin (TAT‐1, Merck) and rat α‐mouse LAMP1 (1DB4, DSHB), were diluted in blocking buffer (PBS, 1% BSA) and incubated overnight at 4˚C in a humidified chamber before washing extensively in blocking buffer. Samples were incubated with goat secondary antibodies (α‐rat IgG AF568 and α‐mouse IgG AF488) and 1:1000 DAPI for 45 min, washed extensively in blocking buffer, and mounted with coverslips using Prolong Gold or Diamond anti‐fade reagents (Invitrogen). Z‐stack series of 0.2 μm intervals were acquired with the ×100 oil objective (numerical aperture 1.45) on an Andor revolution Spinning disk confocal microscope using IQ3 (Andor) software. Settings were kept consistent within an experiment.

### 4.11 Image analysis and quantitation using IMARIS

Image analysis was performed using Imaris software (Bitplane). Images were batch converted into Imaris files. The Display Adjustment tool was used to achieve an appropriate fluorescent signal to avoid oversaturation. Images were exported at a resolution of 300ppx. Granule count per cell and granule diameter were determined in ImageJ v1.52 (NIH). The image z‐stack was used to determine the z‐slice showing the largest diameter of each granule which was then measured using the line tool. Diameter measurements for each granule were recorded for within an individual cell, and this information was then exported to excel. The 3D tool was used to help identify individual granules (using the assumption that granules would largely appear spherical).

### 4.12 Electron microscopy

A total of 2×10^6^ CTLs per sample were washed twice in prewarmed CTL culture medium and resuspended at 1 × 10^6^ cells/ml. Samples were fixed in 1.5% glutaraldehyde/2% paraformaldehyde then washed and post‐fixed (1 h) in 0.1% osmium tetroxide (TAAB laboratories) followed with 0.5% Uranyl Acetate for 16 h at 4C, then processed for EPON embedding. Thin (50‐100nm) sections were stained with lead citrate and analyzed using and viewed on an FEI Tecnai G2 Spirit BioTWIN transmission EM (Eindhoven) and images captured using a Gatan 4K US1000 CCD camera and FEI TIA software and false‐colored using Adobe Photoshop CS6.

### 4.13 Proteomics sample preparation

Cell pellets were lysed in 400 μl lysis buffer (4% SDS, 50 mM TEAB pH 8.5, 10 mM TCEP). Lysates were boiled and sonicated with a BioRuptor (30 cycles: 30 s on, 30 s off) before alkylation with 20 mM iodoacetamide for 1 hour at room temperature in the dark. Lysates were subjected to the SP3 procedure for protein clean‐up [37] before elution into digest buffer (0.1% SDS, 50 mM TEAB pH 8.5, 1mM CaCl_2_) and digested with LysC and Trypsin, each at a 1:50 (enzyme:protein) ratio. TMT labeling and peptide clean‐up were performed as described previously [38]. After labeling samples were eluted into 2% DMSO in water, combined, and dried in vacuo. Samples were then fractionated using off‐line high pH reverse phase chromatography: samples were loaded onto a 4.6 × 250 mm XbridgeTM BEH130 C18 column with 3.5 μm particles (Waters). Using a Dionex BioRS system, the samples were separated using a 25‐min multistep gradient of solvents A (10 mM formate at pH 9 in 2% acetonitrile) and B (10 mM ammonium formate pH 9 in 80% acetonitrile), at a flow rate of 1 mL/min. Peptides were consolidated into 24 fractions which were dried then dissolved in 5% formic acid and analyzed by LC‐MS.

### 4.14 Liquid chromatography–electrospray tandem mass spectrometry analysis (LC–ES‐MS/MS)

1 μg per fraction was analyzed using an Orbitrap Fusion Tribrid mass spectrometer (Thermo Scientific) equipped with a Dionex ultra high‐pressure liquid chromatography system (nano RSLC). RP‐LC was performed using a Dionex RSLC nano HPLC (Thermo Scientific). Peptides were injected onto a 75 μm × 2 cm PepMap‐C18 pre‐column and resolved on a 75 μm × 50 cm RP‐ C18 EASY‐Spray temperature‐controlled integrated column‐emitter (Thermo) using a four‐hour multistep gradient from 5% B to 35% B with a constant flow of 200 nL/min. The mobile phases were: 2% ACN incorporating 0.1% FA (Solvent A) and 80% ACN incorporating 0.1% FA (Solvent B). The spray was initiated by applying 2.5 kV to the EASY‐Spray emitter and the data were acquired under the control of Xcalibur software in a data‐dependent mode using top speed and 4 s duration per cycle, the survey scan is acquired in the Orbitrap covering the m/z range from 400 to 1400 Th with a mass resolution of 120,000 and an automatic gain control (AGC) target of 2.0 e5 ions. The most intense ions were selected for fragmentation using CID in the ion trap with 30% CID collision energy and an isolation window of 1.6 Th. The AGC target was set to 1.0 e4 with a maximum injection time of 70 ms and a dynamic exclusion of 80 s. During the MS3 analysis for more accurate TMT quantifications, 10 fragment ions were co‐isolated using synchronous precursor selection using a window of 2 Th and further fragmented using HCD collision energy of 55%. The fragments were then analyzed in the Orbitrap with a resolution of 60,000. The AGC target was set to 1.0 e5 and the maximum injection time was set to 300 ms.

### 4.15 Processing and analysis of proteomics data

The data were processed, searched, and quantified with the MaxQuant software package, version 1.5.8.3, Proteins and peptides were identified using the UniProt mouse database (SwissProt and Trembl) and the contaminants database integrated in MaxQuant using the Andromeda search engine [39, 40] with the following search parameters: carbamidomethylation of cysteine and TMT modification on peptide N‐termini and lysine side chains were fixed modifications, while methionine oxidation, acetylation of N‐termini of proteins were selected as variable modifications. The false discovery rate was set to 1% for positive identification at the protein and PSM level. The data set was filtered to remove proteins categorized as “contaminants,” “reverse,” and “only identified by site.” Copy numbers were calculated as described [41] after allocating the summed MS1 intensities to the different experimental conditions according to their fractional MS3 reporter intensities. The accuracy of quantification was established using the following guidelines: proteins categorized as high accuracy had more than 8 unique and razor peptides and a ratio for unique/unique + razor greater than or equal to 0.75, proteins categorized as medium accuracy had at least 3 unique and razor peptides and a ratio for unique/unique + razor greater than or equal to 0.5, any proteins below these thresholds were classified as low accuracy.

### 4.16 Statistics and calculations for proteomics data

*p*‐Values were calculated via a two‐tailed, unequal variance *t*‐test on log normalized data. Elements with *p*‐values ≤ 0.05 were considered significant. Fold change thresholds were established using two methods. Method 1 established a fold change cut‐off ≥ 1.5 or ≤ 0.67. Method 2 used the SD of the log_2_‐fold change. A cut‐off was then set as two standard deviations from the mean log_2_ fold change, allowing one to focus on those proteins showing the greatest change in expression. The mass of individual proteins was estimated using the following formula: CN × MW/N_A_ = protein mass (g/cell) where CN is protein copy number, MW is the protein molecular weight in Daltons, and N_A_ is Avogadro's Constant.

### 4.17 Data presentation

Prism 8 (GraphPad Software) was used to present data graphically and generate standard deviations. Student's *t*‐tests were used to calculate *p*‐values.

## Conflict of interest

The authors declare no commercial or financial interest.

## Author contributions

P.R.B. performed all experimental work and analysis except for mass cytometry from ACR; J.C.S. performed TEM; AJD performed pERK immunoblots; J.H. and D.A.C. performed proteomics.

5

### 5.2 Peer review

The peer review history for this article is available at https://publons.com/publon/10.1002/eji.202249797

**Abbreviations**
CTL: cytotoxic T Lymphocyte
TEM: transmission electron microscopy

## Supporting information

Supplemental Information

## Data Availability

Proteomics data is available on the PRIDE database https://www.ebi.ac.uk/pride/archive/projects/PXD036457.
