# Supplementary material for: Super‐killer CTLs are generated by single gene deletion of Bach2
Source: Eur J Immunol. 2022 Sep 26;52(11):1776–88. doi: 10.1002/eji.202249797 (PMC9828676; doi:10.1002/eji.202249797)
Supplement: Supplementary file 1 — Supplemental Information [file EJI-52-1776-s001.pdf]

Supplemental Information

Supplemental Figure 1: Imaging-based cytotoxicity assay showing percentage target lysis over time from WT and BACH2-deficient CTLs, at a 10:1 (CTL:target) ratio. Error bars show standard deviation of values from 4 mice for each of WT and BACH2-deficient CTLs stimulated from splenocytes (> 70% CD8+) or CD8+ enriched populations (>90% CD8+).

Figure S1

A

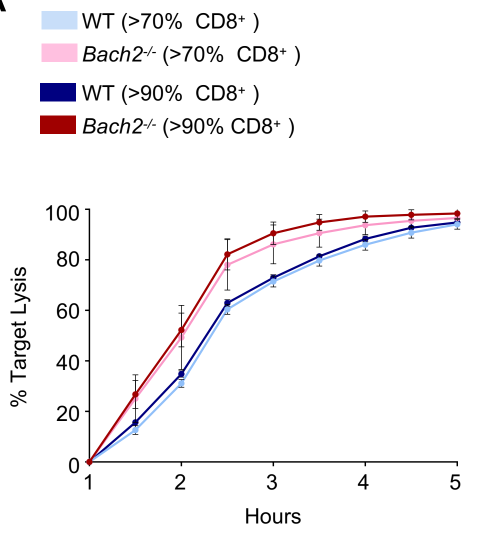

WT (>70% CD8+)
Bach2-/- (>70% CD8+)
WT (>90% CD8+)
Bach2-/- (>90% CD8+)
% Target Lysis
100
80
60
40
20
0
1
2
3
4
5
Hours

Supplemental Figure 2: Results of a differential abundance analysis comparing splenocytes from *Bach2*$^{-/-}$ and WT mice. **A** As Figure 2A for all splenocytes. Colour indicates change in abundance of cells with a given phenotype in *Bach2*$^{-/-}$ versus WT splenocytes (red, increased; blue, decreased). Clusters of cell types manually annotated based on marker expression depicted in B. **B** Median intensity of each mass cytometry marker in each phenotypic population, used to construct the tSNE in A. Labels include metal tag and antibody target.

Figure S2

A

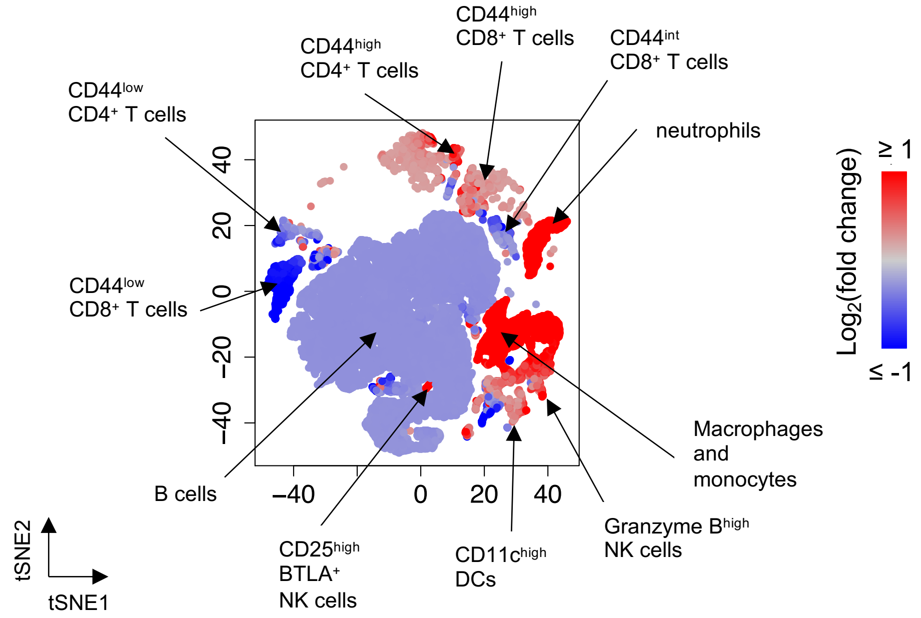
CD44high
CD4+ T cells
CD44high
CD8+ T cells
CD44int
CD8+ T cells
neutrophils
CD44low
CD4+ T cells
CD44low
CD8+ T cells
B cells
CD25high
BTLA+
NK cells
CD11chigh
DCs
Granzyme Bhigh
NK cells
Macrophages
and
monocytes
Log2(fold change)
≥ 1
≤ -1
tSNE1
tSNE2
40
20
0
−20
−40
−40
0
20
40

B

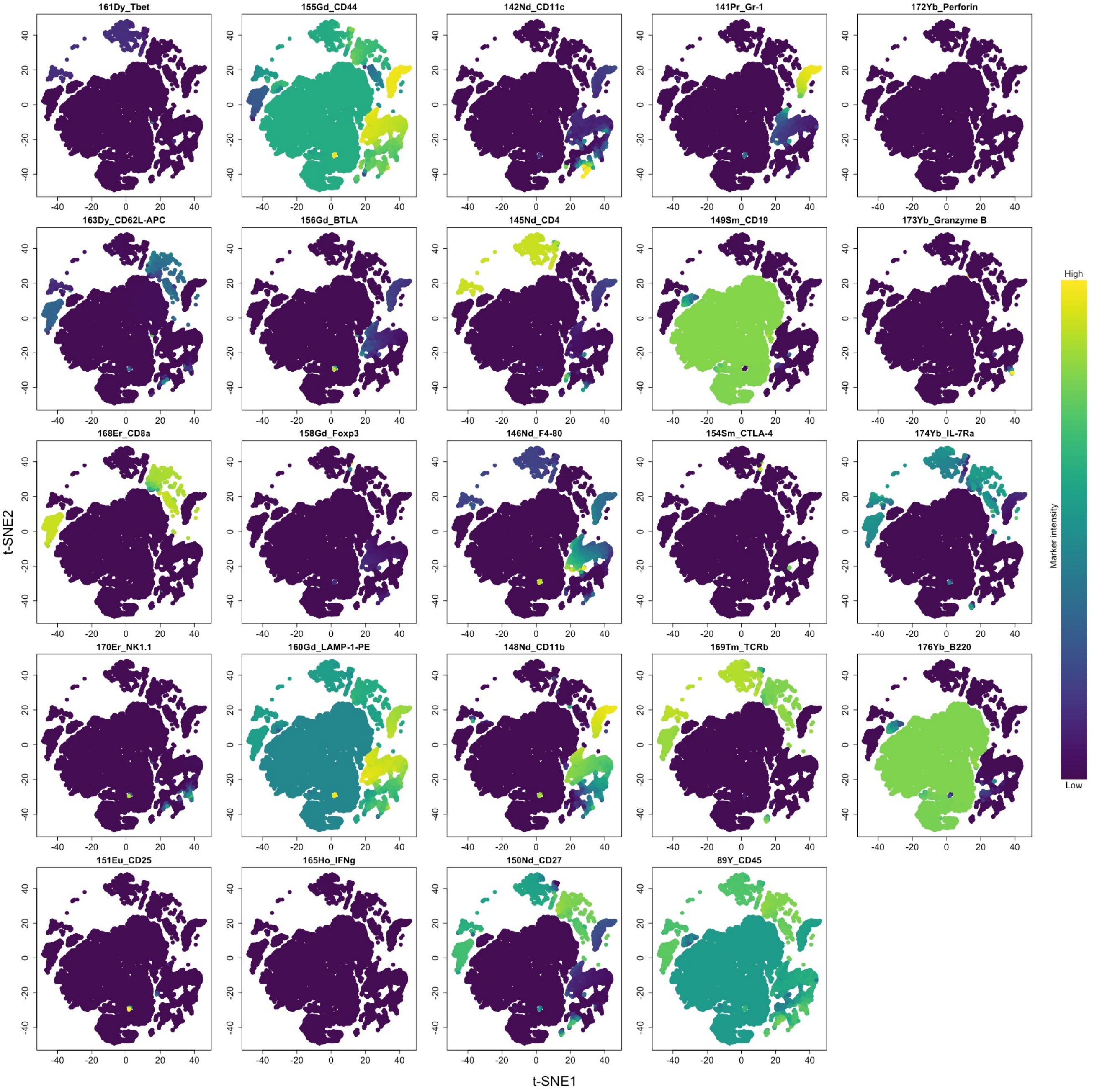
161Dy_Tbet
155Gd_CD44
142Nd_CD11c
141Pr_Gr-1
172Yb_Perforin
163Dy_CD62L-APC
156Gd_BTLA
145Nd_CD4
149Sm_CD19
173Yb_Granzyme B
168Er_CD8a
158Gd_Foxp3
146Nd_F4-80
154Sm_CTLA-4
174Yb_IL-7Ra
170Er_NK1.1
160Gd_LAMP-1-PE
148Nd_CD11b
169Tm_TCRb
176Yb_B220
151Eu_CD25
165Ho_IFNg
150Nd_CD27
89Y_CD45
High
Marker intensity
Low
t-SNE2
t-SNE1

Supplemental Figure 3: Expression of all proteins used to define differentially abundant populations from Figure 2A.

Figure S3

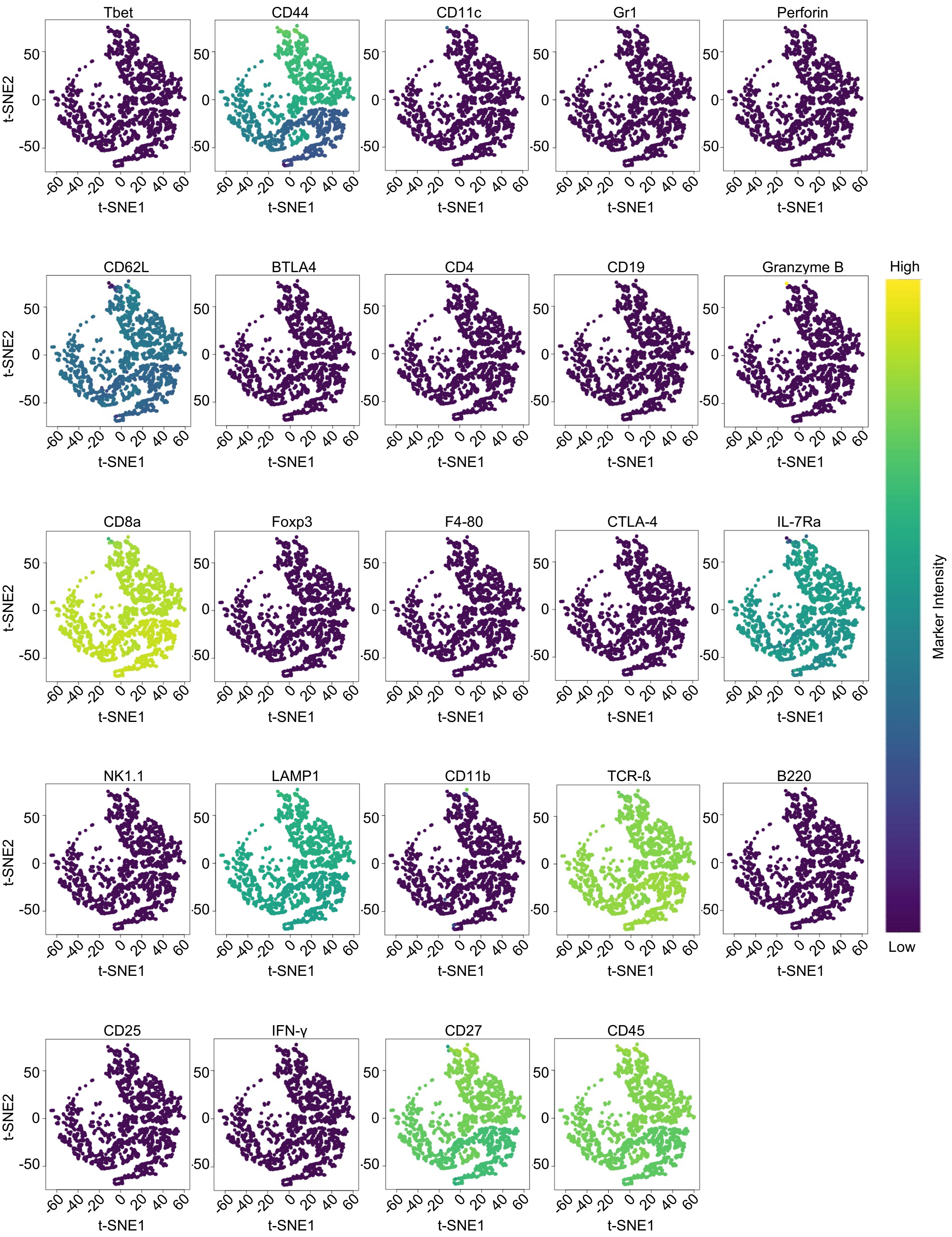

Tbet
CD44
CD11c
Gr1
Perforin
CD62L
BTLA4
CD4
CD19
Granzyme B
CD8a
Foxp3
F4-80
CTLA-4
IL-7Ra
NK1.1
LAMP1
CD11b
TCR-ß
B220
CD25
IFN-γ
CD27
CD45
t-SNE1
t-SNE2
High
Low
Marker Intensity

Supplemental Figure 4: Alternative representation of differentially abundant populations from Figure 2A. Significantly differentially abundant populations were filtered for those with $\log_2$(fold-change) greater than 1 or less than -1. Expression of markers defining these populations were used to hierarchically cluster the populations and are depicted in the yellow-purple colouring of the heatmap. The extent of differential abundance is indicated in the $\log_2$ (fold-change) sidebar, where bright red indicates the greatest increase and bright blue the greatest decrease in abundance in BACH2-deficient compared with WT cells.

Figure S4

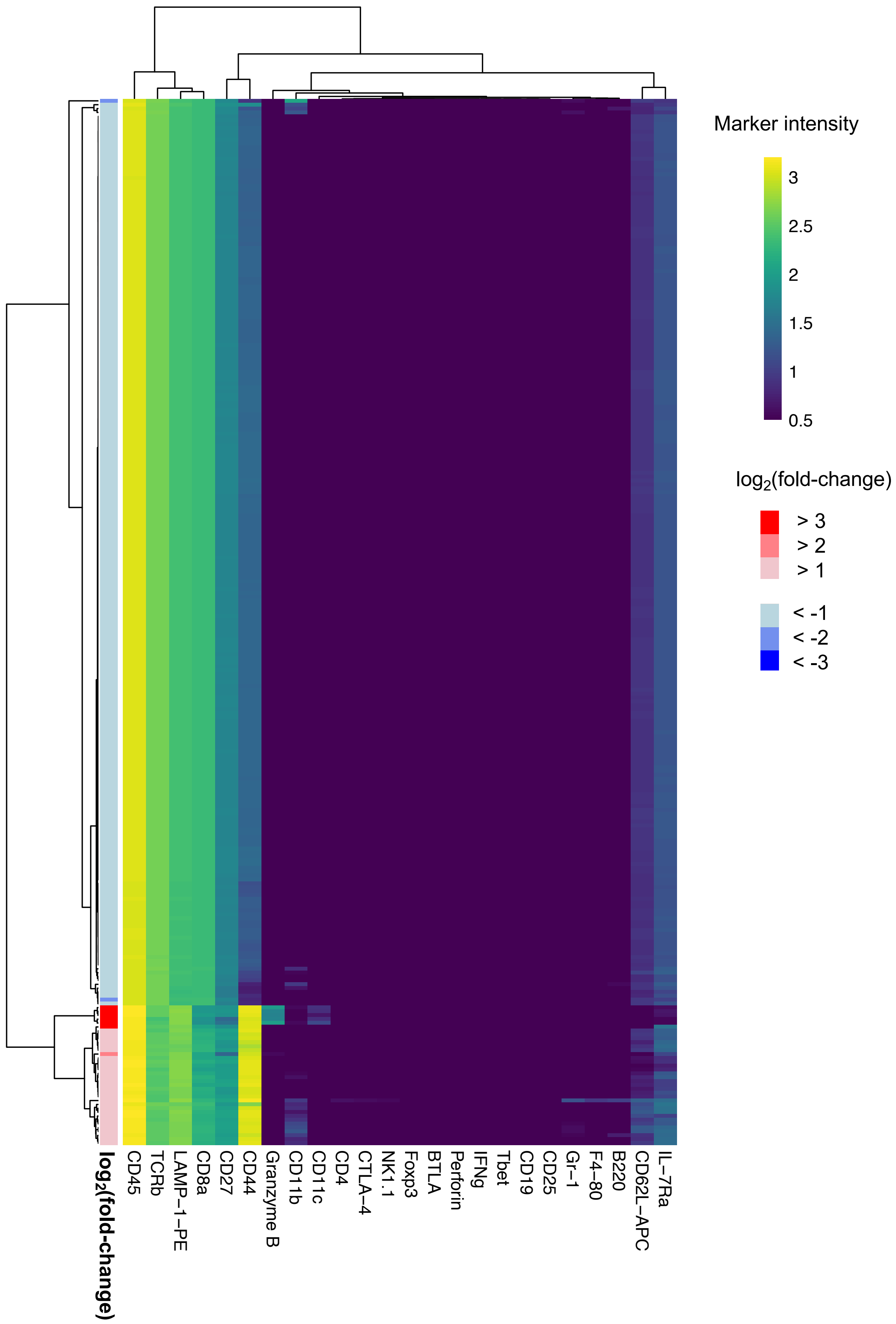

Marker intensity
3
2.5
2
1.5
1
0.5
log2(fold-change)
> 3
> 2
> 1
< -1
< -2
< -3
log2(fold-change)
CD45
TCRb
LAMP−1−PE
CD8a
CD27
CD44
Granzyme B
CD11b
CD11c
CD4
CTLA−4
NK1.1
Foxp3
BTLA
Perforin
IFNg
Tbet
CD19
CD25
Gr−1
F4−80
B220
CD62L−APC
IL−7Ra

Supplemental Figure 5: **A** As Supplemental Figure 2A for day 8 post stimulation. **B** Percentage of $CD8^+$ cells within single, live, $CD45^+$ $TCR\beta^+$ cells. P-value from Student's t test. **C** Median intensity of each mass cytometry marker in each phenotypic population, used to construct the tSNE in A.

Figure S5

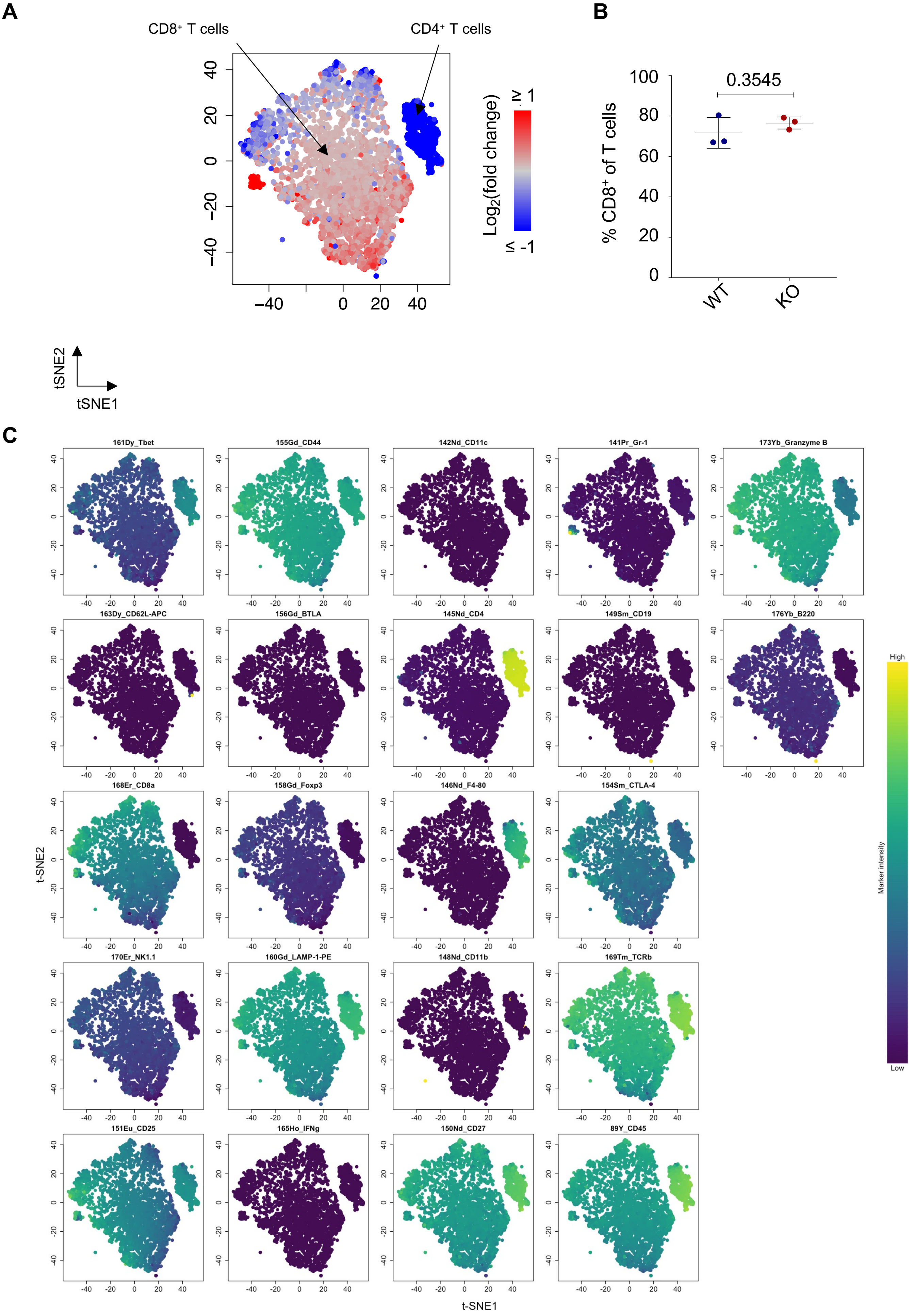

A
CD8⁺ T cells
CD4⁺ T cells
Log2(fold change)
≥ 1
≤ -1
tSNE2
tSNE1
B
% CD8⁺ of T cells
0.3545
WT
KO
C
161Dy_Tbet
155Gd_CD44
142Nd_CD11c
141Pr_Gr-1
173Yb_Granzyme B
163Dy_CD62L-APC
156Gd_BTLA
145Nd_CD4
149Sm_CD19
176Yb_B220
168Er_CD8a
158Gd_Foxp3
146Nd_F4-80
154Sm_CTLA-4
170Er_NK1.1
160Gd_LAMP-1-PE
148Nd_CD11b
169Tm_TCRb
151Eu_CD25
165Ho_IFNg
150Nd_CD27
89Y_CD45
t-SNE2
t-SNE1
High
Marker intensity
Low

Supplemental Figure 6: Expression of all proteins used to define differentially abundant populations from Figure 2F.

Figure S6

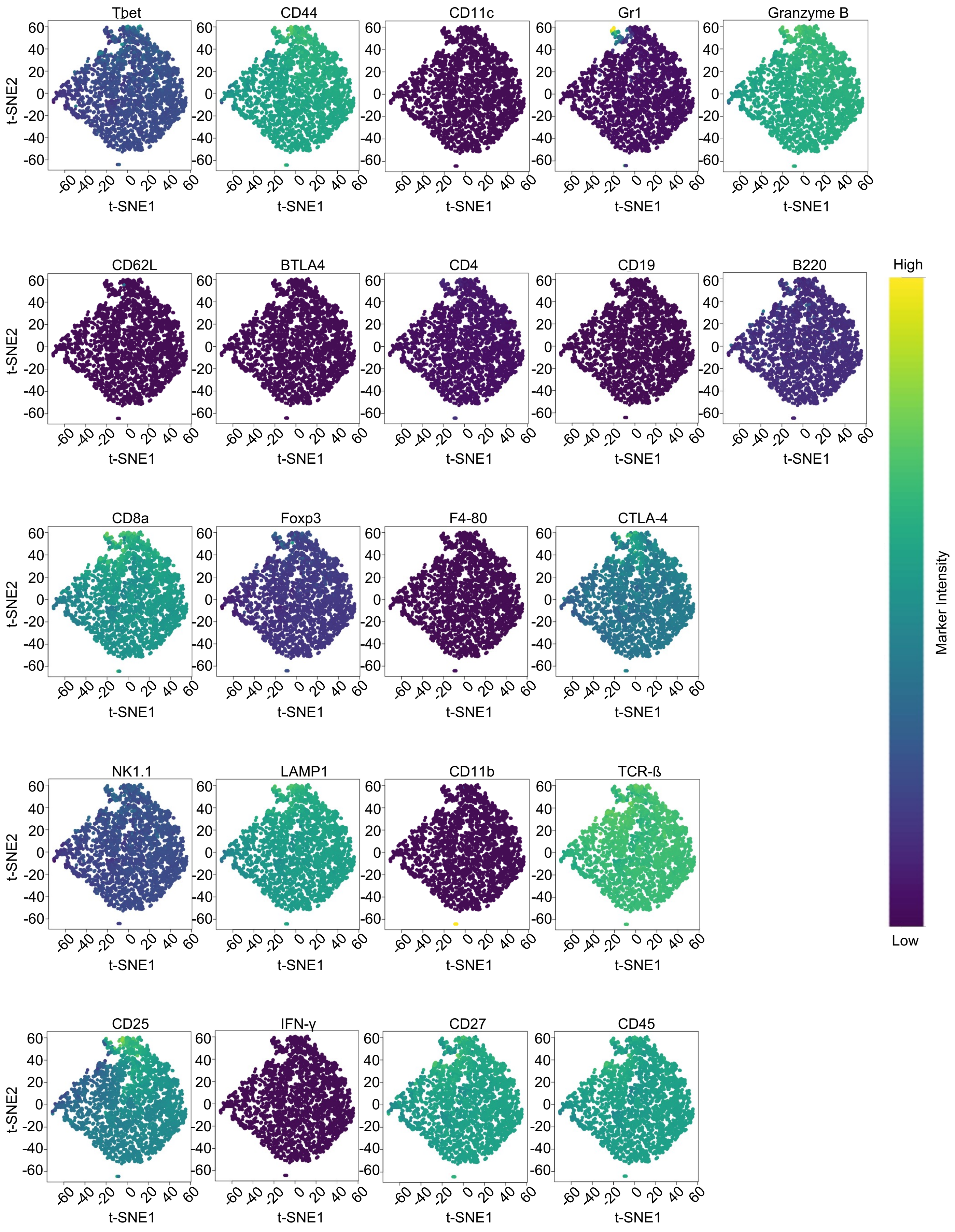

Tbet
CD44
CD11c
Gr1
Granzyme B
CD62L
BTLA4
CD4
CD19
B220
CD8a
Foxp3
F4-80
CTLA-4
NK1.1
LAMP1
CD11b
TCR-ß
CD25
IFN-γ
CD27
CD45
t-SNE1
t-SNE2
High
Low
Marker Intensity

Supplemental Figure 7: Alternative representation of differentially abundant populations from Figure 2F. Significantly differentially abundant populations were filtered for those with log_2(fold-change) greater than 1 or less than -1. Expression of markers defining these populations were used to hierarchically cluster the populations and are depicted in the yellow-purple colouring of the heatmap. The extent of differential abundance is indicated in the log_2(fold-change) sidebar, where bright red indicates the greatest increase and bright blue the greatest decrease in abundance in BACH2-deficient compared with WT cells.

Figure S7

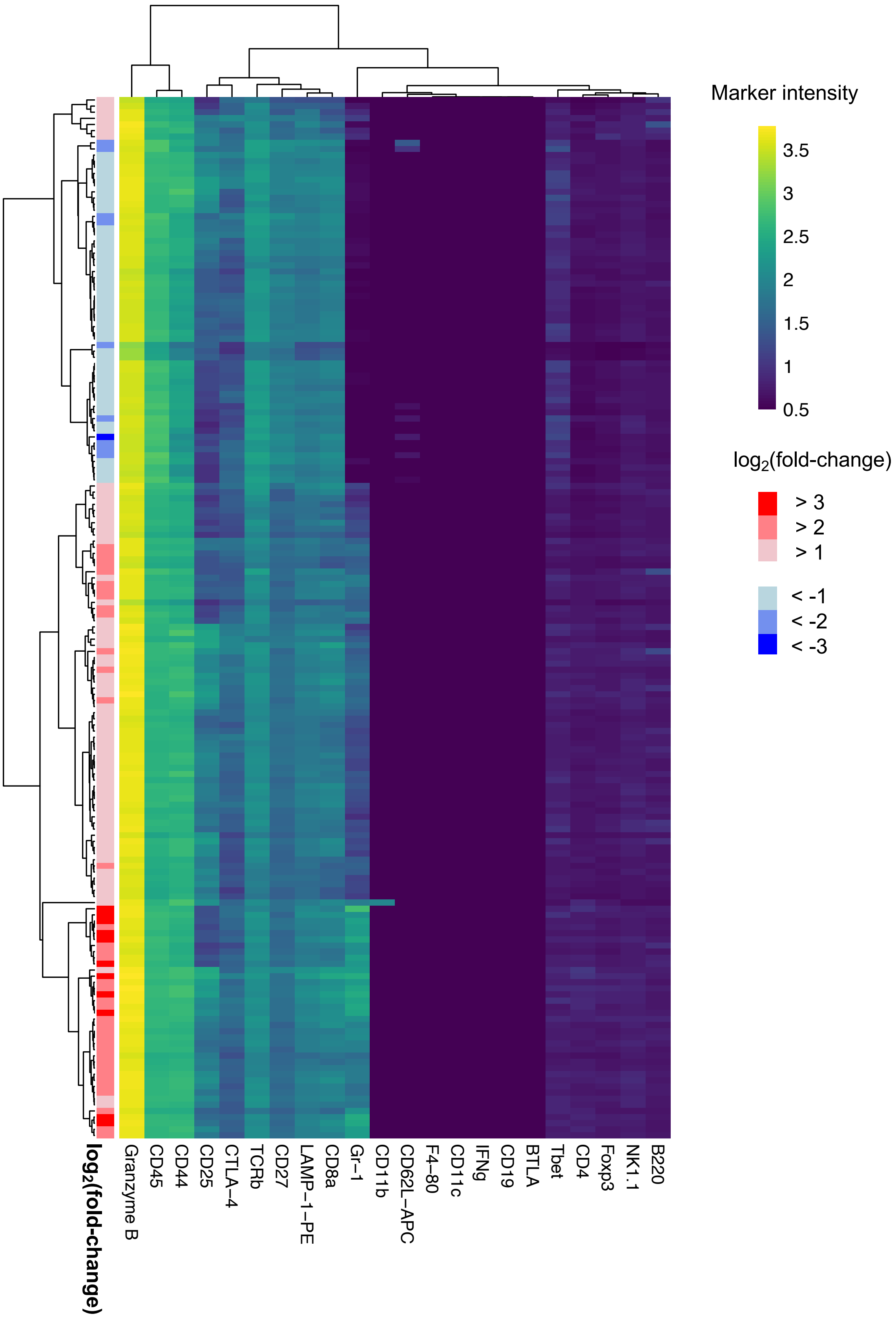

Marker intensity
3.5
3
2.5
2
1.5
1
0.5
log2(fold-change)
> 3
> 2
> 1
< -1
< -2
< -3
log2(fold-change)
Granzyme B
CD45
CD44
CD25
CTLA−4
TCRb
CD27
LAMP−1−PE
CD8a
Gr−1
CD11b
CD62L−APC
F4−80
CD11c
IFNg
CD19
BTLA
Tbet
CD4
Foxp3
NK1.1
B220

Supplemental Table 1: Mass cytometry antibodies

Antibody supplier	Custom conjugation	Label	Target	Antibody Clone
Surface staining primary				
Fluidigm	0	174Yb	CD127 (IL-7Ra)	A7R34
Fluidigm	0	156Gd	CD272 (BTLA)	6F7
Fluidigm	0	150Nd	CD27	LG.3A10
Fluidigm	0	169Tm	TCRb	H57-597
Fluidigm	0	170Er	CD161 (NK1.1)	PK136
Fluidigm	0	168Er	CD8a	53-6.7
Biolegend	1	155Gd	CD44	IM7
Fluidigm	0	151Eu	CD25 (IL-2R)	3C7
Fluidigm	0	89Y	CD45	30-F11
Fluidigm	0	146Nd	F4/80	BM8
Fluidigm	0	145Nd	CD4	RM4-5
Fluidigm	0	149Sm	CD19	6D5
Fluidigm	0	176Yb	CD45R (B220)	RA3-6B2
Fluidigm	0	148Nd	CD11b (Mac-1)	M1/70
Fluidigm	0	142Nd	CD11c	N418
Fluidigm	0	141Pr	Ly-6G/C (Gr-1)	RB6-8C5
Biolegend		APC	CD62L	MEL-14
Surface staining secondary				
Fluidigm	0	163Dy	APC	APC003
Intracellular staining primary				
Fluidigm	0	154Sm	CD152 (CTLA-4)	UC10-4B9
Fluidigm	0	172Yb	Perforin	OMAK-D
Fluidigm	0	173Yb	Granzyme B	GB11
Fluidigm	0	158Gd	Foxp3	FJK-16s
Fluidigm	0	161Dy	Tbet	4B10
Fluidigm	0	165Ho	IFNg	XMG1.2
eBioscience		PE	CD107A (LAMP-1)	eBio1D4B
Intracellular staining secondary				
Biolegend	1	160Gd	PE	PE001
